# Supplementary material for: Diversity and function of soluble heterodisulfide reductases in methane-metabolizing archaea
Source: Microbiol Spectr. 2025 Mar 25;13(5):e03238-24. doi: 10.1128/spectrum.03238-24 (PMC12054007; doi:10.1128/spectrum.03238-24)

**Figure S1. Multiple sequence alignment of HdrA types and their core sequence region.** Multiple sequence alignment of 4 representative sequences (Type I A1, Type Ia A2-1, Type II A3, and Type III A9), along with the representative core region sequence of the Type I A1 after trimming. Red boxes indicate crucial amino acid residues involved in FAD binding, while blue boxes mark the essential iron-sulfur cluster-binding residues located within the thioredoxin reductase domain.


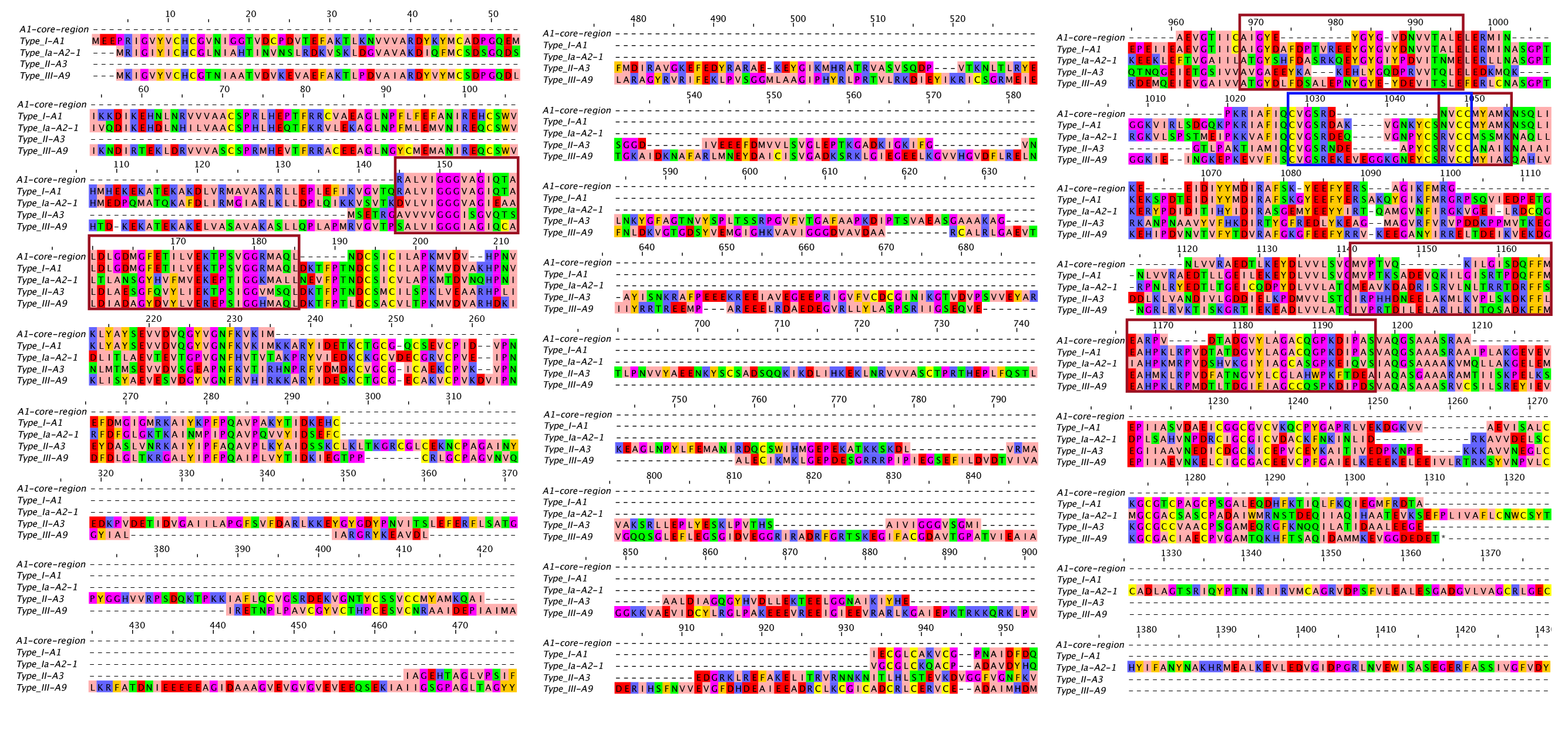


**Figure S2. Phylogeny of the core regions in HdrA proteins.** HdrA types are colored according to Figure 2 in the phylogenetic tree. Black dots on the phylogenetic branches represent bootstrap support values ranging from 0.7 to 1.


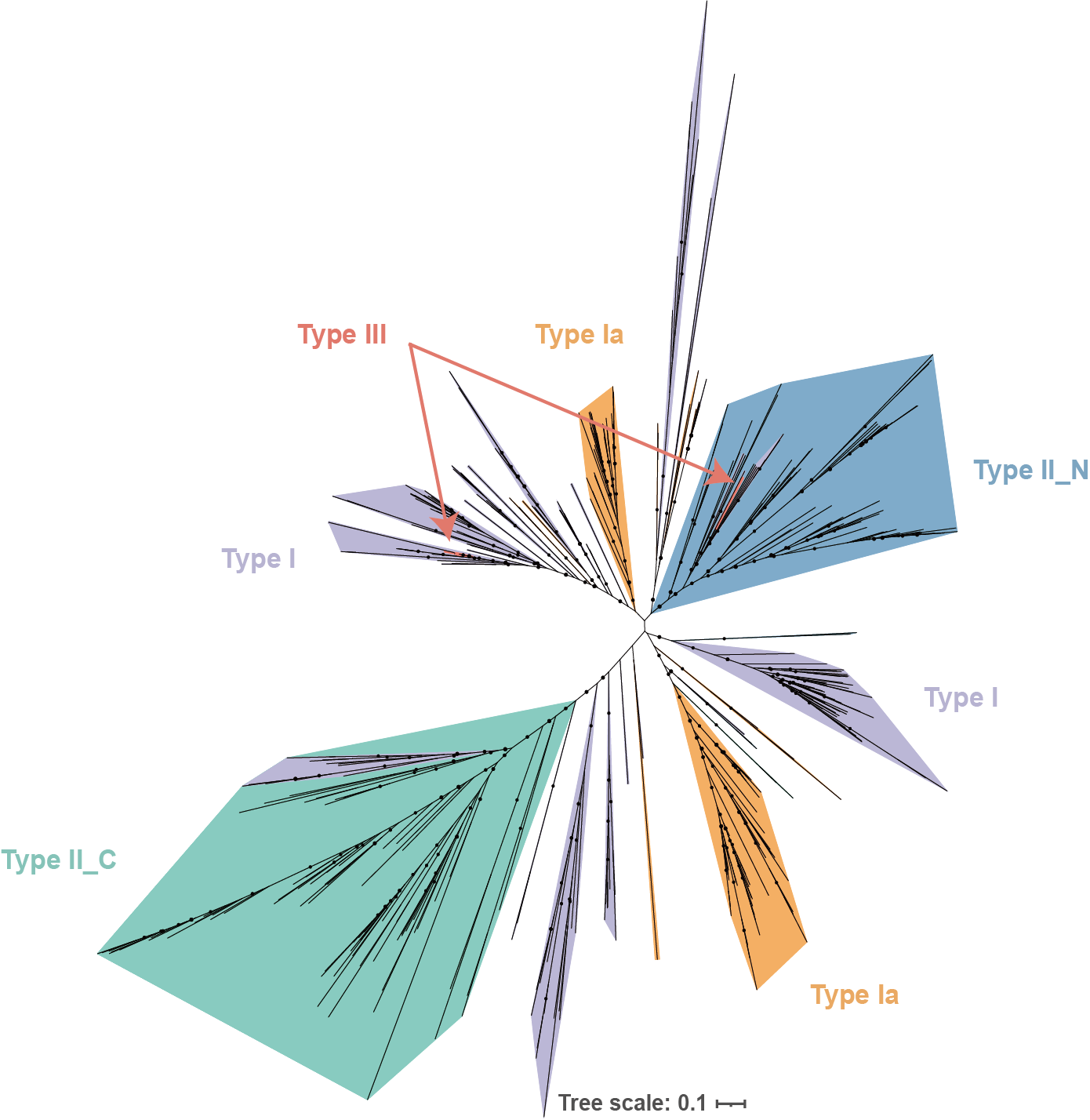


**Fig S3. Analysis of unknown molybdopterin oxidoreductase family proteins in gene clusters of Type II HdrA.** (A) Sequence alignment of unknown clade of molybdopterin oxidoreductase family (Molybdop) proteins with FwdB from *Methanothermobacter wolfeii*, FhcB from *Methylorubrum extorquens*, and FdhA from *Methanospirillum hungatei* JF1, highlighting the key amino acid residue cysteine for cofactor coordination is missing in FhcB and the unknown Molybdop proteins. (B-G) Comparison of characterized protein structures of FwdB from *Methanothermobacter wolfeii*, FhcB from *Methylorubrum extorquens*, FdhA from *Methanospirillum hungatei* JF1, and predicted structures of unknown Molybdop proteins from *Methanolobus profundi*, ANME-2c UBA203, and *Methanoperedens nitroreducens*. The presence of cysteine (red) in FwdB and FdhA enables coordination of the Mo/W-bisPGD cofactor, while the absence of cysteine in FhcB combined with the addition of proline and phenylalaline (red) in the active site prevent the Mo/W-bisPGD) cofactor from forming.


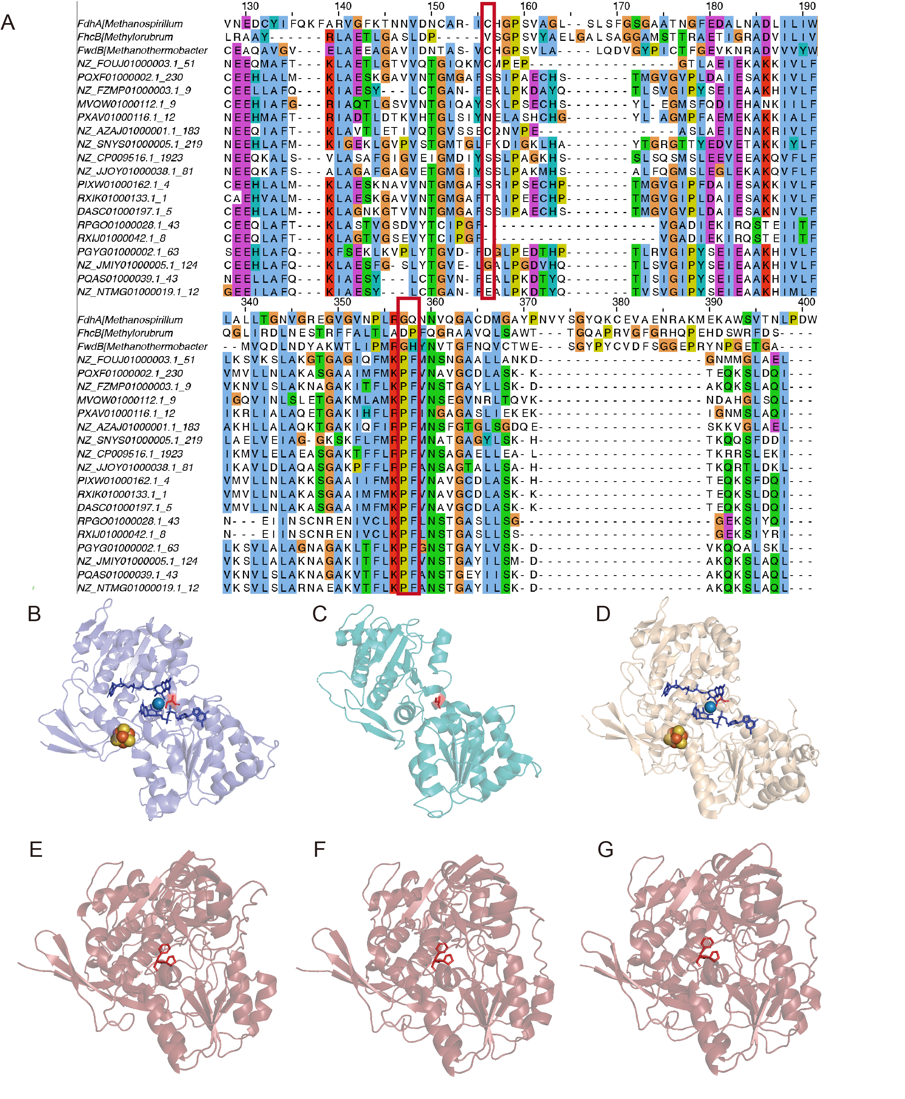


**Fig S4. Structural comparison of FdhB proteins.** (A) Characterized structure of the F420-dependent formate dehydrogenase beta subunit (FdhB) for F_420_H_2_ reaction from *Methanospirillum hungatei* JF-1. (B) Predicted structure of FdhB in the gene cluster of Type II HdrA from *Methanolobus profundi*. The blue sticks represent an unexpected [4Fe-4S] cluster with 3 cysteine (Cys) and 1 histidine (His) ligation, which forms the converging point of two electron transfer pathways in the FdhAB-MvhD complex.


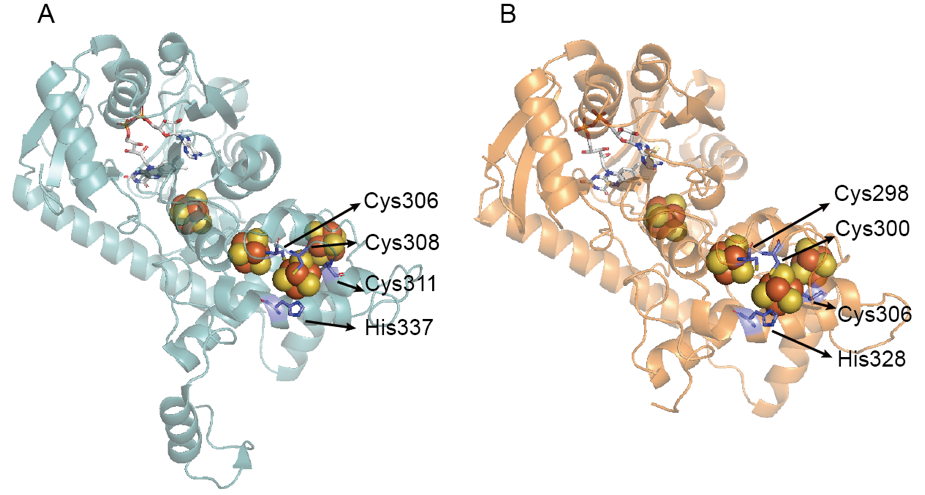


**Figure S5. Gene counts per genome for heterodisulfide reductase subunits.** Subunits of the soluble heterodisulfide reductase (*hdrA*, *hdrB*, *hdrC*) and membrane heterodisulfide reductase (*hdrD* and *hdrE*) in various groups of methane- and alkane-metabolizing archaea. The gene count for *hdrA* is substantially higher than the other Hdr subunits.

**Figure S6. Workflow of the research methodology in this study.** The blue boxes represent the collecting genomes of methane- and alkane-metabolizing archaea phase. The green and yellow boxes represent the identification and classification of HdrA phases: green for HdrA identification and yellow for HdrA classification. The purple and orange boxes illustrate the sequence and structural analyses, covering phylogenetic and evolutionary analysis (purple) and functional prediction (orange).


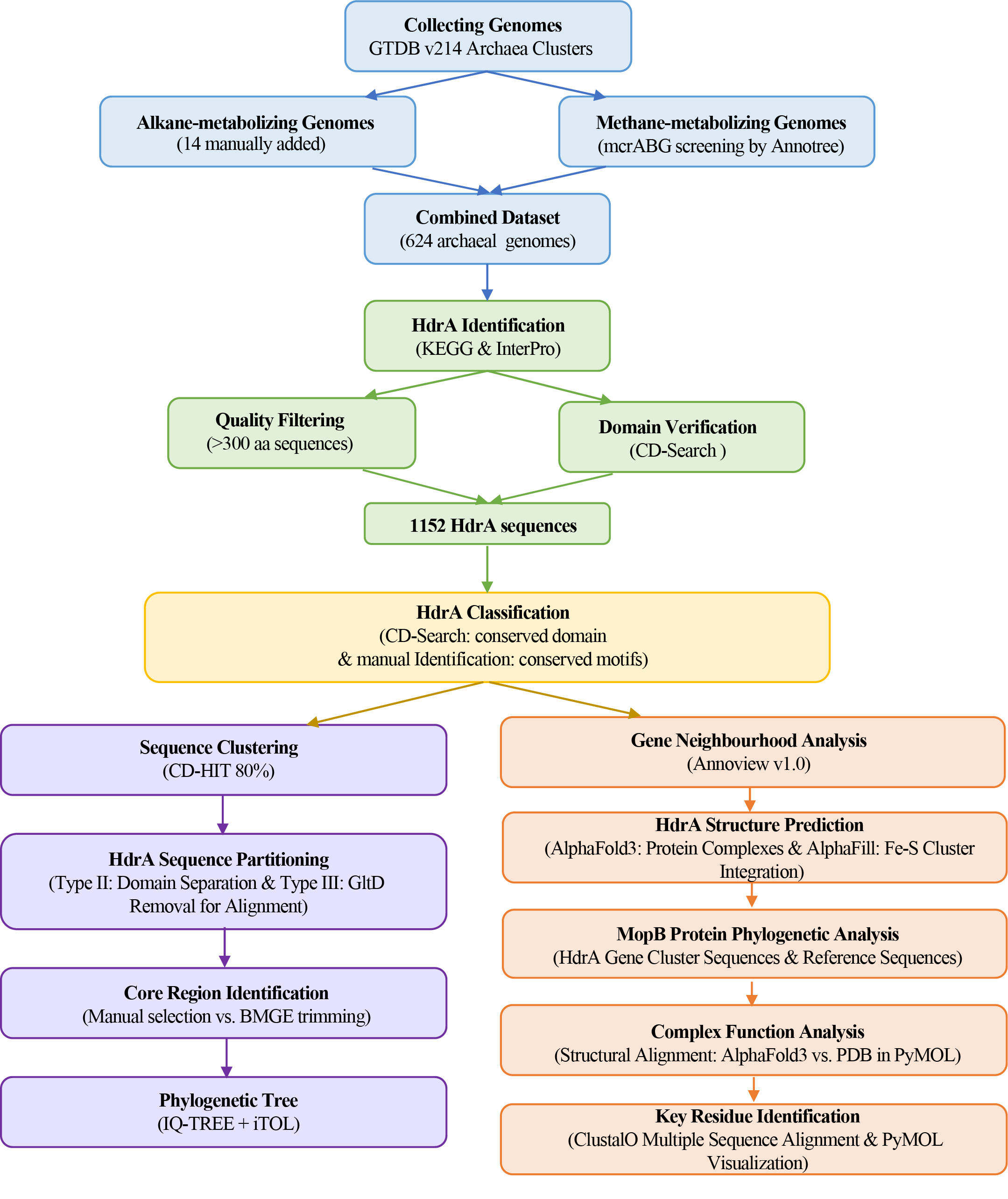

Supplement: Supplemental figures — Fig. S1 to S6. [file spectrum.03238-24-s0001.docx]
